# A Novel, Comprehensive A129 Mouse Model for Investigating Dengue Vaccines and Evaluating Pathogenesis

**DOI:** 10.3390/vaccines11121857

**Published:** 2023-12-15

**Authors:** Mya Myat Ngwe Tun, Khine Mya Nwe, Jean Claude Balingit, Yuki Takamatsu, Shingo Inoue, Basu Dev Pandey, Takeshi Urano, Michinori Kohara, Kyoko Tsukiyama-Kohara, Kouichi Morita

**Affiliations:** 1 Department of Tropical Viral Vaccine Development, Institute of Tropical Medicine, Nagasaki University, Nagasaki 852-8523, Japan; jcpbalingit@gmail.com (J.C.B.); yukiti@nagasaki-u.ac.jp (Y.T.); 2 Department of Virology, Institute of Tropical Medicine, Nagasaki University, Nagasaki 852-8523, Japan; drkhinemyanwe@gmail.com; 3 Center for Vaccines and Therapeutic Antibodies for Emerging Infectious Diseases, Shimane University, Izumo 690-8504, Japan; turano@med.shimane-u.ac.jp; 4 Kenya Research Station, Institute of Tropical Medicine, Nagasaki University, Nagasaki 852-8523, Japan; pampanga@nagasaki-u.ac.jp; 5 Dejima Infectious Diseases Research Alliance, Nagasaki University, Nagasaki 852-8523, Japan; drbasupandey@gmail.com; 6 Department of Diseases and Infection, Tokyo Metropolitan Institute of Medical Science, Tokyo 156-0057, Japan; kohara-mc@igakuken.or.jp; 7 Transboundary Animal Diseases Centre, Joint Faculty of Veterinary Medicine, Kagoshima University, Kagoshima 890-0065, Japan; kkohara@vet.kagoshima-u.ac.jp

**Keywords:** A129 mouse, Dengvaxia vaccine, dengue virus, neutralization antibody, viral loads

## Abstract

In search of a mouse model for use in evaluating dengue vaccines, we assessed A129 mice that lacked IFN-α/β receptors, rendering them susceptible to dengue virus (DENV) infection. To our knowledge, no reports have evaluated dengue vaccine efficiency using A129 mice. A129 mice were given a single intraperitoneal (IP) or subcutaneous (SC) injection of the vaccine, Dengvaxia. After 14 days of immunization via the IP or SC injection of Dengvaxia, the A129 mice exhibited notably elevated levels of anti-DENV immunoglobulin G and neutralizing antibodies (NAb) targeting all four DENV serotypes, with DENV-4 displaying the highest NAb levels. After challenge with DENV-2, Dengvaxia and mock-immunized mice survived, while only the mock group exhibited signs of morbidity. Viral genome levels in the serum and tissues (excluding the brain) were considerably lower in the immunized mice compared to those in the mock group. The SC administration of Dengvaxia resulted in lower viremia levels than IP administration did. Therefore, given that A129 mice manifest dengue-related morbidity, including viremia in the serum and other tissues, these mice represent a valuable model for investigating novel dengue vaccines and antiviral drugs and for exploring dengue pathogenesis.

## 1. Introduction

Dengue is the world’s most prevalent mosquito-borne viral disease and has a considerable economic burden in tropical and sub-tropical countries. In recent years, there has been an increase in the incidence of dengue infection. Globally each year, there are approximately 400 million new infections in total, of which approximately 100 million are clinically apparent [1]. Dengue virus (DENV) of the Flaviviridae family is the causative agent of dengue and it has four serotypes (DENV-1, DENV-2, DENV-3, and DENV-4) that are antigenically distinct [2]. The virus consists of an 11-kilobase, single-stranded RNA molecule that encodes three structural and seven non-structural (NS) proteins. The structural proteins are the capsid (C), precursor membrane (PrM), and envelope (E) proteins and the seven NS proteins are NS1, NS2A, NS2B, NS3, NS4A, NS4B, and NS5. A person infected with DENV may be asymptomatic or symptomatic. Symptomatic infection may be self-limiting dengue fever (DF) or severe dengue hemorrhagic fever (DHF), which may potentially progress to fatal dengue shock syndrome (DSS) [3]. Severe DHF and DSS are commonly observed in children and in adolescents under 15 years of age [4]. Manifestations of severe infection include shock, hemorrhage, plasma leakage, major organ failure, encephalitis, and myocarditis, and they vary according to viral and host factors as well as antibody-dependent enhancement (ADE), the interplay of which is still not well understood [5]. ADE occurs because pre-existing subneutralizing antibodies and the infecting DENV form complexes that bind to Fc receptor-bearing cells, leading to increased virus uptake and replication [6].

Two major hypotheses have been proposed to explain the marked differences in disease severity that can result from DENV infection. One hypothesis suggests that viral mutation and evolutionary forces drive increased virulence and that those viruses require no pre-existing antibodies to cause severe primary infection; another suggests that pre-existing immunity to DENV may increase the severity of secondary DENV infection through ADE [7,8,9]. No approved antiviral agents exist for the prevention and treatment of DENV infection; however, several vaccine candidates are currently in clinical development. Dengue vaccines, including live-attenuated, inactivated, recombinant subunit, viral vector, and DNA vaccines have been investigated. Dengvaxia, the first dengue vaccine to be licensed, is a live-attenuated, tetravalent vaccine that includes genes expressing prM and E structural proteins from all four DENV serotypes and NS protein genes from the yellow fever virus vaccine strain 17D [10,11]. However, this vaccine failed to elicit a balanced immune response against all four DENV serotypes and instead increased the risk of severe disease in DENV-seronegative vaccine recipients [11]. Because of safety concerns in young children, it led to the recommendation that the vaccine be given only to those who are nine years of age and older. The World Health Organization recommended that the vaccine be used only in countries with high endemicity [12].

There is a considerable need to investigate new treatment options, including novel and promising vaccine candidates, anti-viral drugs, and biotherapeutics that could address the limitations of current options. Two other tetravalent live-attenuated vaccine candidates, namely TAK-003 by Takeda and TV003 by the National Institute of Allergy and Infectious Diseases, have completed phase 3 clinical trials [13]. Additionally, Takeda’s live-attenuated tetravalent dengue vaccine candidate (TAK-003) based on the DENV-2 backbone is under evaluation in a long-term clinical trial across eight dengue-endemic countries [14]. The two major challenges in dengue vaccine development are ADE and a lack of accessible, cheap, and sensitive animal models that mimic the human immune response. Common, wild-type laboratory mice are naturally resistant to DENV and do not develop clinical signs of infection because the virus does not inhibit murine IFN signaling as it does for human interferon (IFN) signaling [15].

Despite this limitation, several immunocompetent mice and immunocompromised mouse models have been investigated [16,17]. Because DENV infection inhibits IFN production and signaling, mice with deficient IFN responses are useful models of DENV infection [18]. AG129 mice are deficient in IFN type I (IFN-α/β) and type II (IFN-γ) receptors and have been widely used to study the pathogenesis of the four DENV serotypes, DENV immunity, and the potential of antiviral drugs [19]. Additionally, AG129 mice have been used to investigate the immunogenicity of tetravalent dengue vaccines and their protective efficacy following lethal DENV challenge [10,20]. However, not only did the antibodies produced by Dengvaxia fail to protect against lethal DENV challenge, but they also elicited an ADE in DENV infection in AG129 mice [21]. In this study, we developed a novel mouse model that is susceptible to DENV infection and is suitable for Dengvaxia vaccine evaluation. We used A129 mice that lacked IFN-α/β receptors. This study showed that these mice produced neutralizing antibodies (NAb) following Dengvaxia administration and had significant protection against subsequent DENV challenge.

## 2. Materials and Methods

### 2.1. Virus and Cells

For virus challenge in mice, DENV-2 N-36 (MN 083232) was used in this study. This virus strain was isolated from an acute-phase serum sample (three days from the start of illness) of a six-year-old girl who had severe, fatal dengue in Sri Lanka in 2017 [22]. The virus strains used for the serological tests, namely in an in-house DENV immunoglobulin G (IgG) indirect enzyme-linked immunosorbent assay (ELISA) and focus reduction neutralization assay, were as follows: DENV-1 (99St12A, genotype IV), DENV-2 (00St22A, Asian genotype II), DENV-3 (SLMC-50, genotype I), and DENV-4 (SLMC-318, genotype I) [23]. The DENV viruses were propagated in the *Aedes albopictus* mosquito cell line C6/36, and the culture fluid was used after a few passages. The C6/36 cells were cultured at 28 °C in minimum essential medium (MEM) supplemented with 2% fetal calf serum (FCS). The DENV stock strains were stored at −80 °C prior to use.

### 2.2. Immunizations and Virus Challenge of Mice

**Figure 1:**
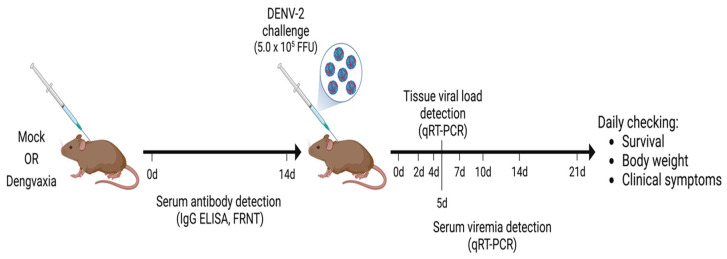
Schematic diagram of the generation of immune serum, and the challenge with DENV-2 involved in the A129 mouse model. A129 mice were immunized either intraperitoneally or subcutaneously with a single dose of Dengvaxia or PBS. After vaccination, serum samples were collected on days 0 and 14 for antibody detection and mice were then challenged with the DENV-2 virus. Other activities are indicated in the diagram above.

The tetravalent live-attenuated vaccine Dengvaxia was obtained as a lyophilized powder and was reconstituted in 0.5 mL of phosphate-buffered saline (PBS) solution. After reconstitution, the vaccine contained a 4.5–6.0 log_10_ CCID_50_ (=50% cell culture infectious dose) of each of the four DENV serotypes. The A129 mice were purchased from B & K Universal Limited. The mice were mated in the Nagasaki University animal facility. Groups (n = 10) of 4–6-week-old A129 mice were given a single subcutaneous (SC) or intraperitoneal (IP) injection of Dengvaxia (0.1 mL/mouse). In addition, a group of A129 mice (n = 10) were subcutaneously injected with PBS solution to serve as mock-immunized controls. Serum samples from the Dengvaxia and control groups were collected on day 0 (before mock or Dengvaxia immunization) and on day 14 after immunization to measure the levels of dengue-specific antibodies. On day 14, all mice received a DENV-2 (5 × 10^5^ focus-forming unit/mouse) challenge via SC injection (0.2 mL). For the morbidity and mortality studies after virus challenge, 5 mice from each of the groups above were weighed daily for 21 days and observed for clinical signs of dengue. Serum was collected on days 0, 2, 4, 7, 10, and 14 to measure the viremia levels via a quantitative real-time reverse transcription polymerase chain reaction (qRT–PCR). To measure the viremia levels in the organs and tissues after virus challenge, the other 5 mice from each of the groups above were sacrificed on day 5. The thymus, lung, heart, liver, spleen, stomach, small intestine, large intestine, kidney, brain, spinal cord, and muscle tissue were collected. Figure 1 shows a schematic diagram of the procedure described here.

### 2.3. Measurement of DENV-Specific Antibodies

Blood samples extracted on days 0 and 14 after Dengvaxia and mock immunization were centrifuged, and serum samples were collected. A DENV-IgG indirect enzyme-linked immunosorbent assay (ELISA; Nunc, Roskilde, Denmark) was performed using procedures modified from previous studies [23]. Ninety-six-well ELISA plates were coated with purified DENV at 250 ng/well and incubated at 4 °C overnight. The plates were blocked with Block Ace (Kac, Tokyo, Japan) for 1 h at room temperature and then washed with PBS-Tween 20 (PBS-T). The experimental and positive control serum samples diluted at 1:100 in PBS-T were added to the designated wells and plates were incubated for 1 h at 37 °C, after which the plates were washed. A horseradish peroxidase-conjugated anti-mouse IgG antibody (American Qualex, San Clemente, CA, USA) diluted at 1:2000 was added to the wells. Plates were incubated for 1 h at 37 °C and then washed. The reaction was allowed to develop with o-phenylenediamine dihydrochloride (Sigma, St. Louis, MO, USA) in substrate buffer and 0.03% hydrogen peroxide solution via incubation in the dark for 30 min. The reaction was stopped with the addition of 1 N sulfuric acid, and the optical density at 492 nm was read.

### 2.4. Measurement of DENV Neutralization Antibodies

The neutralizing activity of DENV-specific mouse antibodies was determined by conducting a 50% focus reduction neutralization (FRNT50) assay [23]. Serum samples collected from mice on days 14 after Dengvaxia immunization were heat-inactivated at 56 °C for 30 min before the assay. These samples, initially diluted to 1:10, were further serially diluted in a 2-fold ratio. Then, each of these samples was mixed with an equal volume of DENV that contained 40 focus-forming units (FFU). After incubation at 37 °C for 1 h, the serum and virus mixtures were transferred to 96-well plates containing confluent Vero cell monolayers. After incubation at 37 °C for 1 h, cells were overlaid with 2% FCS MEM containing 1% methylcellulose 4000 (Wako, Osako, Japan). The plates were then incubated at 37 °C with 5% CO_2_. After 3 days, the cells were fixed, blocked, and permeabilized as described previously. Viral foci were detected by immunostaining the cells with anti-DENV serum pooled from patients infected with dengue, peroxidase-conjugated anti-human IgG (American Qualex, CA, USA), and 3,3-diaminobenzidine substrate (Wako, Osaka, Japan). The endpoint serum dilution that provided a ≥50% reduction compared to the mean of the control wells (the negative control that contained DENV virus and MEM) was considered the FRNT50 titer. A neutralization titer of ≥10 was considered a confirmed DENV infection. Mouse serum with an FRNT_50_ titer of 640 against 4 serotypes of DENV was used as the positive control serum. Figure S1A shows a representative positive control well with the complete inhibition of the formation of infected foci. A representative negative control well yielded about 40 foci (Figure S1B).

### 2.5. Quantification of Viremia Levels via qRT–PCR

To quantify the DENV genome RNA levels in serum and tissues, we extracted the total RNA using Isogen II (Nippon Gene, Tokyo, Japan) in accordance with the manufacturer’s instructions. A volume of 5 µL of RNA was used for qRT–PCR, and the amplification of the envelope gene was performed by using 20 µL of a reaction mixture comprising 5 μL of Taqman master mix, 9 µL of nuclease water, 0.3 µL of 100 pmol forward and reverse primers, and 0.4 µL of a probe with DENV serotype-specific primers of TaqMan Fast Virus 1-Step Master Mix (Life Technologies, Carlsbad, CA, USA), following a previously described protocol [24]. qRT–PCR was performed using the Quant Studio 7 Flex real-time PCR system (Applied Biosystems, Waltham, MA, USA) with the following program: 50 °C for 5 min, 95 °C for 20 s, 40 cycles of 95 °C for 3 s, and 60 °C for 30 s. Viral genome levels were expressed as copies/mL if RNA was from serum, or as copies/gram if it was from tissues.

### 2.6. Statistical Analysis

Data were analyzed using SPSS for Windows (version 16.0; IBM Corp., Armonk, NY, USA) and GraphPad Prism 9 (GraphPad Software, Inc., San Diego, CA, USA). All comparisons among the three groups were performed using the Kruskal–Wallis test. The Mann–Whitney U test assessed significant differences between groups in the levels of virus copies in the serum and tissues. Student’s *t* test was used to analyze differences in body weight change between the two groups of mice. A *p* value of <0.05 was considered statistically significant.

## 3. Results

### 3.1. Humoral Immune Response in A129 Mice after Immunization with Dengvaxia

**Figure 2:**
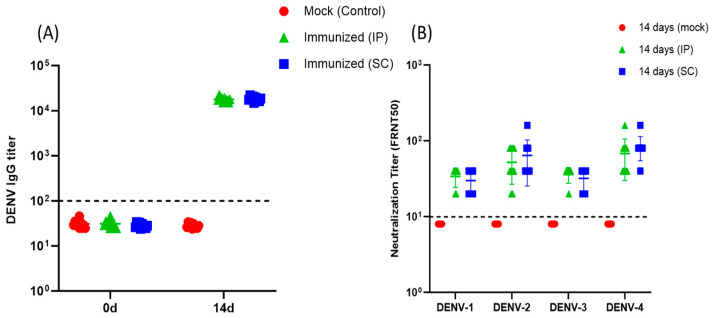
(**A**) Anti-DENV IgG responses to mock-immunized (n = 10), intraperitoneally immunized (n = 10), and subcutaneously immunized (n = 10) A129 mice at days 0 and 14. Immunized mice were given a single dose of Dengvaxia at day 0. (**B**) Neutralization antibody titers against four DENV serotypes on the 14th day after immunization of mice with Dengvaxia via intraperitoneal (n = 10) and subcutaneous (n = 10) administration. A comparison between two groups was performed using the Mann–Whitney U test. All comparisons among the neutralization antibody titers against four serotypes of DENV were performed using the Kruskal–Wallis test. *p*-value < 0.05 was considered statistically significant.

Fourteen days after A129 mice received a single IP or SC immunization with Dengvaxia, an antibody response was noted with significantly higher anti-DENV IgG levels compared to those on day 0 (Figure 2A). There were no significant differences in antibody levels between mice that received IP and the SC administration doses. No detectable anti-DENV IgG antibodies were seen in the control mice at days 0 and 14 after mock immunization. All Dengvaxia-immunized mice regardless of immunization had NAb against all four DENV serotypes at day 14 (Figure 2B). For those mice with IP Dengvaxia immunization, the mean NAb titers against DENV-1, DENV-2, DENV-3, and DENV-4 were 34, 52, 36, and 68., respectively. For those with SC Dengvaxia immunization, the respective mean NAb titers against DENV-1, DENV-2, DENV-3, and DENV-4 were 30, 64, 32, and 84. Dengvaxia elicited the highest NAb titers against DENV-4 relative to the other serotypes regardless of the administration route. No significant differences in NAb titers were found between mice that received Dengvaxia via IP and those that received it via SC.

### 3.2. Morbidity and Mortality in Immunized A129 Mice Following DENV-2 Infection

**Figure 3:**
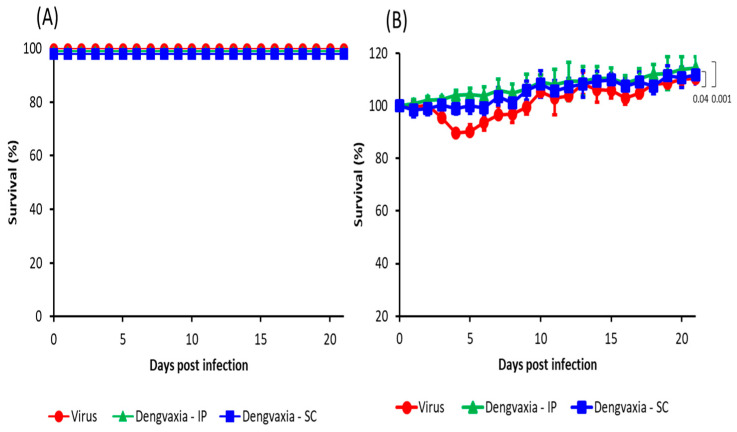
(**A**) Survival rate and (**B**) body weight change (%) in the three groups of A129 mice after challenge with DENV-2 (5 × 10^5^ FFU/mouse). The three groups were Dengvaxia IP (n = 5), Dengvaxia SC (n = 5), and the virus control (n = 5). The body weights of the mice were monitored daily at the same time and recorded as a percentage of initial body weight. Student’s *t* test was used to analyze the body weight change between the two groups of mice. A *p* value < 0.05 was considered statistically significant.

Observations of the disease course following DENV-2 challenge in the A129 mice that received single IP or SC Dengvaxia immunization and in those that received mock immunization showed that all immunized and mock-immunized mice (except those mice that were sacrificed on the 5th day after virus challenge for the detection of tissue viremia) were alive up to 21 days post-infection (pi) (Figure 3A). The mock-immunized mice began to exhibit clinical signs, such as weight loss and slow movement at 3–6 days pi; however, these mice recovered, and their weight increased at 7 days pi (Figure 3B). The immunized mice exhibited no change in body weight, and as a result, there were significant differences in the weight change between mock-immunized mice and those receiving Dengvaxia via SC (*p* = 0.04) and IP (*p* = 0.001) routes (Figure 3B).

### 3.3. Viremia in Immunized A129 Mice Following DENV-2 Challenge

**Figure 4:**
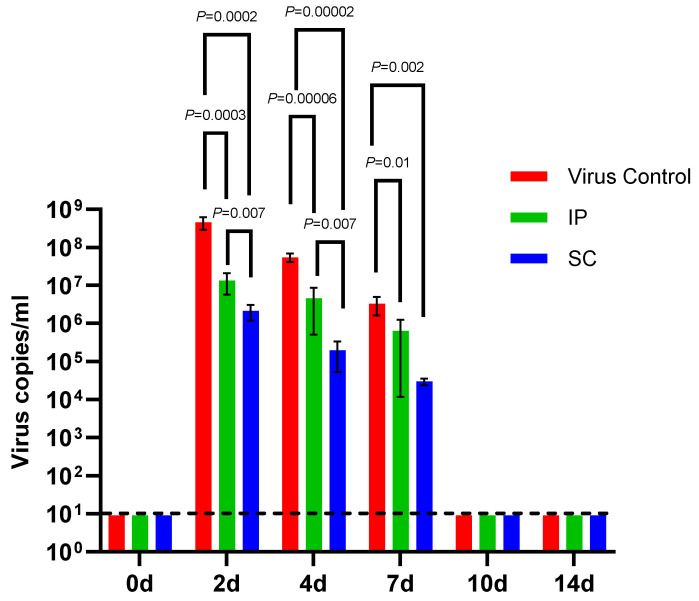
Viremia levels of serum (RNA copies/mL) in individual mice of the three groups of A129 mice after DENV-2 (5 × 10^5^ FFU/mouse) challenge. The three groups were Dengvaxia IP (n = 5), Dengvaxia SC (n = 5), and the virus control (n = 5) groups. A comparison between two groups was performed using the Mann–Whitney U test. A *p*-value < 0.05 is considered statistically significant.

We compared the viral RNA levels in serum from mice that received IP or SC Dengvaxia with those from the mock-immunized mice at 0, 2, 4, 7, 10, and 14 days pi with DENV-2 (Figure 4). Following infection with DENV-2, the mean viral RNA copies/mL in serum from mice that received IP Dengvaxia were significantly lower than that in the mock-immunized mice at 2, 4, and 7 days pi. The mean viral RNA copies/mL in the serum from mice that received SC Dengvaxia was significantly lower than that in the mock-immunized mice at 2, 4, and 7 days pi. Viral RNA levels were the highest 2 days pi in all groups. However, mice that received SC Dengvaxia had significantly lower viremia RNA levels at 2 and 4 days pi than did those that received an IP dose.

### 3.4. Viral Load in Organs and Tissues of Immunized A129 Mice Infected with DENV-2

**Figure 5:**
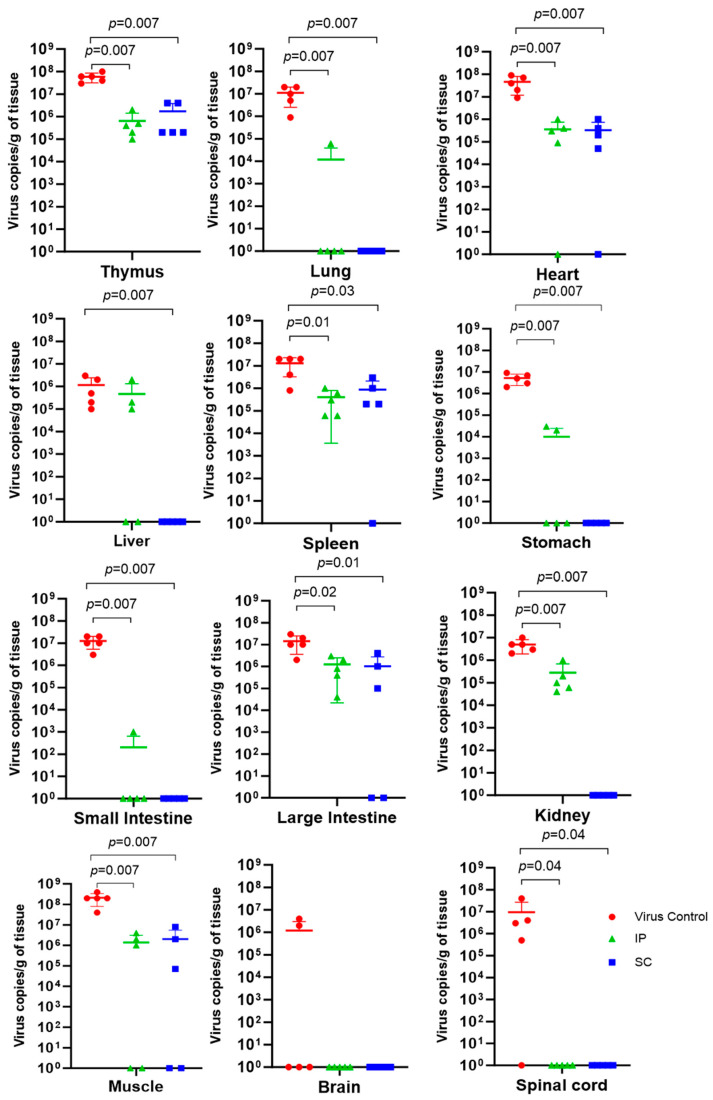
Viral load (RNA copies/g of tissue) in each tissue/organ of individual mice from the three groups of A129 mice 5 days post-infection with DENV-2 (5 × 10^5^ FFU/mouse). The three groups were Dengvaxia IP (n = 5), Dengvaxia SC (n = 5), and the virus control (n = 5) groups. A comparison between two groups was performed using the Mann–Whitney U test. A *p* value < 0.05 was considered statistically significant.

Viral RNA levels in most organs and tissues of DENV-2-challenged Dengvaxia-immunized mice (regardless of the route of infection) were significantly lower from those of DENV-2-challenged mock-immunized mice after 5 days pi (Figure 5). These significantly lower viral RNA levels were observed in the thymus, lung, heart, spleen, stomach, small intestine, large intestine, kidney, muscle, and spinal cord, but not in the liver of the group of mice immunized with Dengvaxia via the SC route and challenged with DENV-2. We noted that viral RNA levels in the brain tissue were detected in the mock-immunized group of mice (two out of five mice), whereas they were not detected in any mice that received Dengvaxia immunization via IP or SC administration. Viral RNA levels in the brain of immunized and mock-immunized mice were not significantly different. Viral RNA levels in the spinal cord were detected in the mock-immunized group of mice (four out of five mice) but were not detected in any of the mice immunized via IP or SC routes.

## 4. Discussion

Based on recombinant DNA technology, several live attenuated vaccines have been produced, such as the chimeric yellow fever 17D virus tetravalent dengue vaccine (Dengvaxia), the recombinant DENV-4 mutant vaccine bearing a 30-nucleotide deletion (TV003/TV005), and the chimeric DENV-2 PDK-53-based tetravalent vaccine DENVvax (Takada 003) [25]. Preclinical studies of Dengvaxia in nonhuman primates (monkeys) of the Takeda 003 vaccine in AG129 mice and nonhuman primates, and of the TV003/TV005 vaccine in nonhuman primates have been performed [20,26]. Nonhuman primates are useful animal models because they have similar immune responses to those of humans. However, they are costly and thus, researchers preferably use mouse models for pre-clinical trials [27].

To develop a comprehensive mouse model for evaluation, we used the Dengvaxia vaccine in this study. We used A129 mice that lacked IFN-α/β receptors as a novel animal model for evaluating dengue pathogenesis and developing novel vaccines. The roles of type I and type II IFN contributions are important for determining viral pathogenic mechanisms. The A129 mouse model has been used for DENV, Japanese encephalitis virus, West Nile virus, Zika virus, yellow fever virus, Mayaro virus, and Chikungunya virus infection [19,28,29,30,31,32]. To our knowledge, no reports have evaluated dengue vaccine efficiency using A129 mice.

A single SC dose of Dengvaxia led to the production of tetravalent dengue antibodies in cynomolgus macaques [33]. Similarly, our study showed that a single IP and SC immunization of A129 mice with Dengvaxia induced both binding DENV IgG antibodies and NAbs against DENV after 14 days. The geometric mean titers of NAb response in monkeys were 113 for DENV-1, 718 for DENV-2, 285 for DENV-3, and 1140 for DENV-4 after 1-month immunization with a single SC dose of Dengvaxia, demonstrating that DENV-4 had a predominant NAb [33]. In addition, the DENV-4 component in Dengvaxia replicates to higher titers in animals and people compared with the other three serotypes studied previously [34]. Thus, in our study, we noted that DENV-4 had a higher NAb compared to DENV-1, DENV-2, and DENV-3 after immunization with Dengvaxia in A129 mice.

In the present study, we used our clinical dengue isolates for neutralization tests owing to the unavailability of Dengvaxia vaccine parent strains (DENV-1: PUO-359/TVP-1140 (ThailandP), DENV-2: PUO-218 (Thailand), DENV-3: PaH881/88 (Thailand), and DENV-4: S1228 (Indonesia)) in our lab [35]. As previously reported, the vaccine stimulated Ab, which that could neutralize DENV-4 genotype II better than it could genotype I [36]. Thus, the low NAb levels we observed in both intraperitoneally and subcutaneously immunized A129 mice may be related to the animal model, immunization time, duration of immunization before challenge with the virus, and the virus strain genotypes used for the neutralization test. Despite the low NAb levels, DENV-2 viremia was significantly reduced in immunized mice in our study. Therefore, not only a humoral immune response (binding Ab IgG and NAbs) but also a cellular response may contribute to viral clearance in A129 mice.

Many studies have reported the role of B cells and T cells in protecting against DENV infection [20,37,38]. Mice lacking IFN-α/β and IFN-γ receptors in the 129 genetic background are highly susceptible to DENV infection [39]. The virus requires multiple passages for adaptation; thus, to induce acute and lethal DENV infection in AG129 mice, we have to inject them with a high dose because they become more resistant to viral challenge with age [19]. Antibodies elicited via Dengvaxia immunization in these mice failed to offer protection against lethal DENV challenge [21]. In humans, dengue severity is likely associated with the viremia level [40]. In our study, infection with a clinical isolate of DENV-2 (5 × 10^5^ FFU SC) in mock-immunized A129 mice was not lethal; however, the mice developed signs of morbidity, including weight loss and high viremia levels in serum and tissues 4–7 days pi. In contrast, these symptoms were not observed following DENV-2 challenge in A129 mice that received Dengvaxia via a single IP or SC injection.

The vaccinated mice had significantly lower viremia levels in their serum and tissues compared with those in the mock-immunized group. However, no significant differences (*p* = 0.4) were observed between the viremia levels in the brains of mock-immunized and immunized mice. Our results are in agreement with those of previous studies in which A129 mice had lower viral RNA in their spinal cords and brains than AG129 mice did, demonstrating the role of IFN signaling in protecting the central nervous system from disease following DENV infection; the A129 mice likely retained sufficient IFN receptors to minimize brain viremia [19]. A previous study on dengue pathogenesis demonstrated that IFN-γ receptor signaling plays a critical role in the host defense against DENV in two distinct phases: a limitation of systemic virus replication in the early stage and a clearance of infection from the CNS in the late stage [19]. In our study, SC and IP administration to the Dengvaxia mice group led to an increased generation of DENV antibodies and lower viremia levels in the serum, tissues, and organs compared to those in the mock-immunized mice group.

There are limitations in our study because we did not measure cellular immune response (levels of associated cytokines, mediators, etc.) after Dengvaxia immunization, whereas we described humoral immune response in A129 mice. Another limitation is that the challenge test was carried out only with DENV-2 infection. For our further studies, we will include measurements of cellular immune response after Dengvaxia administration and challenge the vaccinated mice with the other three serotypes of DENV. Here, we showed that A129 mice may serve as a useful model for evaluating DENV morbidity and viral replication in vivo, thus facilitating both the investigation of mechanisms of dengue pathogenicity and novel vaccines and antiviral drugs.

## Data Availability

Data are contained within the article.

## Supplementary Materials

The following supporting information can be downloaded at https://www.mdpi.com/article/10.3390/vaccines11121857/s1: Figure S1. A representative well of focus reduction neutralization test against DENV on positive control mouse serum (A) which has completely inhibited the formation of infected foci, and negative control (B) which yielded about 40 foci.
