# Supplementary figures and images for: A Novel, Comprehensive A129 Mouse Model for Investigating Dengue Vaccines and Evaluating Pathogenesis
Source: Vaccines (Basel). 2023 Dec 15;11(12):1857. doi: 10.3390/vaccines11121857 (PMC10748371; doi:10.3390/vaccines11121857)

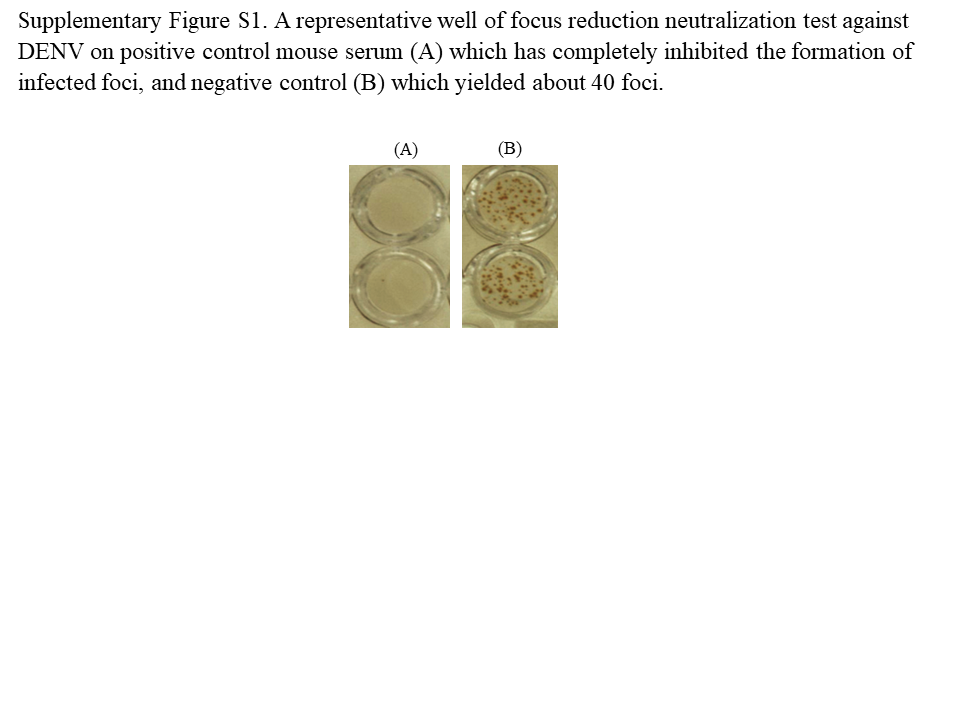

Supplement: Supplementary file 1 [file vaccines-11-01857-s001.zip › vaccines-2711591-supplementary.tif]
